# DNA source and primer choice affect the reliability of metabarcoding for nematode community profiling in agricultural soils

**DOI:** 10.1371/journal.pone.0344569

**Published:** 2026-04-10

**Authors:** Jerry Akanwari, Md Rashedul Islam, Ping Liang, Tahera Sultana

**Affiliations:** 1 Department of Biological Sciences, Brock University, St. Catharines, Ontario Canada; 2 London Research and Development Centre, Agriculture and Agri-Food Canada, Vineland Station, Ontario, Canada; 3 National Microbiology Laboratory, Public Health Agency of Canada, Winnipeg, Manitoba, Canada; University of Limpopo, SOUTH AFRICA

## Abstract

The advent of metabarcoding has greatly advanced our understanding of nematode ecology by overcoming many limitations associated with traditional morphology-based methods. The NF1–18Sr2b (NF1) is the standard and widely used primer pair to assess nematode communities. However, this primer also presents challenges, especially when applied to DNA extracted directly from soil, where it has been shown to underestimate species richness. Consequently, soil DNA extraction (SE) has been viewed as a less attractive option for metabarcoding studies. To explore whether nematode DNA extraction (NE) can serve as a viable option for metabarcoding studies, we compared two degenerate primer pairs, NemF–18Sr2b (NemF) and NemFopt–18Sr2bRopt (NemFopt) to NF1. The study used two DNA sources: NE, and SE derived from 10 g and 1.25 g of dry agricultural soils. Our findings indicate that the NemF and NemFopt primers yielded higher taxonomic resolution and species richness in both SE and NE compared to NF1. Specifically, NF1 detected only 2–4% of nematode sequences in SE and 69% in NE, whereas NemFopt detected the highest proportion, with 100% of nematode sequences in NE and >70% in SE. Although none of the primers amplified all taxa, NF1 was associated with higher undetected taxa. NemFopt in the 10 g SE identified nematode assemblages comparable to those from NE, suggesting that SE can effectively capture nematode communities similar to NE. Nematode community profiles and ecological indices were stable across soil DNA input volumes and DNA sources. Maturity index and enrichment index did not differ significantly between NE and SE. Community similarity across extraction methods varied with primer choice, with NemFopt showing the greatest consistency. Although this study was limited to two soil types, two field sites, and three primer sets, our results suggest that increasing primer specificity can reduce the amount of soil required for metabarcoding and make SE a viable option. Moreover, primer choice, soil type, and DNA source can significantly affect nematode diversity estimates and ecological interpretations, highlighting the need for primer standardization in nematode metabarcoding studies.

## Introduction

Agricultural soils support a wide diversity of organisms, with nematodes ranking among the most abundant and functionally important metazoans [1]. These microscopic organisms inhabit nearly all terrestrial and aquatic environments, including extreme habitats such as Antarctica, the Great Salt Lake and deep subsurface soils [2–4]. Within the food web, nematodes play key ecological roles by contributing to organic matter decomposition, nutrient mineralization, and the suppression of plant diseases [5].

Advances in molecular techniques, particularly metabarcoding, have revolutionized our capacity to study nematode diversity and ecology. The approach offers the possibility of detecting rare species and provides higher taxonomic resolution than traditional morphology-based methods, which are often time-consuming and require taxonomic expertise [6–10]. As a result, metabarcoding has become increasingly attractive and widely adopted tool for assessing soil biodiversity and health [6,9,10].

However, both the DNA source and primer choice can heavily influence nematode metabarcoding results. The most common approach involves isolating nematodes from soil prior to DNA extraction, hereafter referred to as nematode DNA extraction [10,11]. Although nematode DNA extraction has become a standard protocol, it is labour-intensive, often requires large volumes of soil (ideally >200 g), and currently lacks commercial kits specifically optimized for nematode-rich samples [12–14].

Alternatively, extracting DNA directly from soil can reduce processing time, minimize species detection bias associated with traditional extraction protocols, and enable the identification of low-abundance nematodes [15]. Soil DNA extraction is already a popular and well-established approach in studies of fungi and bacterial ecology; however, it has been less widely adopted for nematode metabarcoding [16,17]. The main constraints limiting its acceptance in nematology are the relatively small volumes of soil that most extraction kits can handle and the lack of nematode-specific primers [13,18,19].

The development of metabarcoding has resulted in the design of several primers aimed at characterizing nematode communities [20,21]. Among these, the small-subunit 18S rRNA primer pair NF1–18Sr2b is one of the most widely used markers for profiling nematode communities [6,18,22]. However, the NF1–18Sr2b (NF1) primer set has notable limitations when applied to soil DNA extraction, as it tends to co-amplify non-target sequences from plants, fungi, protists, and other eukaryotes [13,19]. It has been shown to amplify only 4–10% nematode sequences in soil DNA extractions [13,19,23]. The consistently poor nematode recovery from direct soil DNA has impeded the broader adoption of this method for nematode metabarcoding studies.

To optimize the NF1–18Sr2b primer, Sapkota and Nicolaisen (23) designed the NemF–18Sr2b (NemF) primer set by modifying the NF1 primer to better match conserved nematode sites while excluding mismatches at the 3′ end. Subsequently, Waeyenberge, de Sutter (14) introduced degenerate bases into the NemF–18Sr2b primer to improve GC content to develop the NemFopt–18Sr2bRopt (NemFopt) primer set. These optimized primers have demonstrated improved performance, with NemF reported to recover over 74% of nematode sequences from soil-derived DNA samples [23,24].

Despite these advances, no study has directly compared the performance of NF1, NemF, and NemFopt using both nematode DNA and soil DNA extracted from agricultural soils. As a result, it remains unclear whether optimized primers make soil DNA extraction a viable alternative to nematode DNA extraction for community profiling. Prior assessments of soil DNA extraction for nematode metabarcoding have relied largely on a single primer set, which may underestimate the method’s full potential. Primer choice can strongly influence nematode detectability, and extraction efficiency may be further improved through protocol optimization. The development and standardization of soil DNA extraction protocols will improve the comparability and reproducibility of nematode community assessments across ecological studies and facilitate the implementation of global soil biodiversity monitoring programmes. Therefore, we hypothesized that an optimized soil DNA extraction method, when combined with suitable primer sets, could yield nematode community profiles comparable to those obtained from nematode extraction regardless of soil input volume. To address this gap, the present research aims to (i) evaluate the specificity of the NF1, NemF, and NemFopt primers in characterizing nematode communities, and (ii) compare community profiles derived from nematode and soil DNA extractions using different soil types and volumes. The study hypothesizes that the NemF and NemFopt primers will improve nematode detection in soil DNA extractions, and that the best-performing primer will yield community profiles comparable between nematode DNA extraction (NE) and soil DNA extraction (SE). The results of this study are intended to provide guidance on the optimal selection of primers and DNA extraction methods for metabarcoding analyses of nematode communities in agricultural soils.

## Materials and methods

### Site description and soil sampling

Soil samples were collected from experimental fields located at 3Gen Organics Farm (43° 38’ 59.568” N 80° 37’ 14.628” W) and VanMeer Farms Inc. (42° 47’ 19.896” N 80° 37’ 24.888” W) in Southern Ontario, Canada. The soil at 3Gen Organics Farm, referred to as clay soil, consisted of 39% sand, 29% silt, and 32% clay. In contrast, the soil at VanMeer Farms Inc., referred to as sandy soil, comprised 77% sand, 15% silt, and 8% clay. Both sampling sites have been under corn and soybean production integrated with winter cover crops for more than 10 years. Soil sampling was carried out prior to corn harvest.

At each site, four plots (4 m² each) were randomly selected per treatment. From each plot, 10 composite soil cores were collected using a standard 2.5 cm diameter soil probe. All soil samples were placed in labeled polyethylene bags and stored at 4°C until further processing. The samples were homogenized, and two 50 g subsamples were taken from each: one for nematode DNA extraction and the other for soil DNA extraction.

### Nematode DNA extraction (NE)

Nematodes were isolated from 50 g of each soil sample using the sugar flotation and centrifugation method [25]. This method relies on the specific gravity between nematodes and soil particles to isolate both active and inactive nematodes and is widely used in nematology research [6,10]. Extracted nematodes were allowed to settle for 24 hours at 4°C and then concentrated to 1 mL by carefully removing the supernatant with a pipette. The concentrated nematodes were transferred into 2 mL Eppendorf tubes, flash-frozen in liquid nitrogen for 5 seconds, and stored at −20°C until DNA extraction [14]. DNA was extracted using the DNeasy Blood and Tissue Kit (Cat: 69504, Qiagen Inc., Mississauga, ON, Canada) following the manufacturer’s instructions, except that 180 μL of Buffer ATL and 20 μL Proteinase K were added. The mixture was vortexed and incubated overnight at 55°C.

### Soil DNA extraction (SE)

Genomic DNA was extracted from 10 g (SE.10) and 1.25 g (SE.1.25) of dry soil samples using two different kits. The DNeasy PowerMax Soil Kit (Cat: 12988−10, Qiagen Inc., Mississauga, ON, Canada) was used for 10 g samples, while the DNeasy PowerSoil Pro Kit (Cat: 47014, Qiagen Inc., Mississauga, ON, Canada) was used for the 1.25 g samples. These kits were selected based on differences in extraction efficiency, cost, and availability. Prior to extraction, 50 g of soil from each sample was freeze-dried for 48 hours using a Labconco Freeze Dryer (Cat: 700402000, Labconco, Mississauga, ON, Canada) and manually ground with a porcelain mortar and pestle. The 1.25 g extractions were processed using five bead tubes from the DNeasy PowerSoil Pro Kit. The resulting DNA extracts were pooled by combining equal volumes (150 µL) from each tube to create a composite sample. All DNA extractions were performed according to the manufacturer’s instructions.

### Amplicon sequencing

All extracted DNA samples were quantified using a Nano spectrophotometer (Model: DS-11 FX, FroggaBio, Concord, ON, Canada) and stored at −20°C. An average of 20 ng/µL DNA was sent to Génome Québec (Montréal, Québec, Canada) for PCR amplification, indexing, library preparation, and sequencing using the primer sets mentioned in Table 1. PCR amplification was performed in 25 µL reaction volumes using Roche FastStart High-Fidelity polymerase, with an annealing temperature of 53 °C, and 35 amplification cycles. Reactions followed the facility’s validated metabarcoding workflow targeting the 18S rRNA V6–V8 region. Paired-end sequencing (2 × 300 bp) was performed on an Illumina MiSeq v3 platform (Illumina Inc., San Diego, CA, USA). A total of 72 samples were sequenced (2 sites x 4 plots x 3 DNA sources x 3 primer sets).

**Table 1:** Primer pairs used in this study.

Primer	Target region	Nematode specificity and resolution	Forward and reverse sequence	Amplicon size (bp)	Reference
NF1–18Sr2b	18S rRNA (V6 – V8)	Widely used for metabarcoding. Not nematode-specific. Genus to species level resolution	GGTGGTGCATGGCCGTTCTTAGTT / TACAAAGGGCAGGGACGTAAT	200 - 500	Porazinska, Giblin-Davis (22)
NemF–18Sr2b	18S rRNA (V6 – V8)	Optimized degenerate version of the widely used NF1 / 18Sr2b. Genus to species level resolution	GGGGAAGTATGGTTGCAAA / TACAAAGGGCAGGGACGTAAT	300 - 700	Porazinska, Giblin-Davis (22); Sapkota and Nicolaisen (23)
NemFopt–18Sr2bRopt	18S rRNA (V6 – V8)	Optimized degenerate version of NemF / 18Sr2b. Genus to species level resolution	GGGGWAGTATGGTTGCAAA / TGTGTACAAAKGRCAGGGAC	300 - 700	Waeyenberge, de Sutter (14)

### Bioinformatics

Bioinformatics analyses were conducted using the nf-core/ampliseq Nextflow pipeline v23.10.0 [26]. Demultiplexed FASTQ files were imported into the pipeline, and raw read quality reports were generated using FastQC v0.12.1 (Accessed on March 21, 2024, https://www.bioinformatics.babraham.ac.uk/projects/fastqc/). Primer and adaptor sequences were trimmed, and quality filtering was performed using Cutadapt v1.16 [27]. Forward and reverse reads were truncated to 280 bp and 275 bp, respectively, and reads exceeding a maximum expected error threshold of 2 (maxEE = 2) were discarded. The resulting high-quality reads were subsequently processed in QIIME 2 v2024.10 [28]. Operational taxonomic units (OTUs) clustering was performed at 99% sequence identity threshold. Taxonomic assignment was performed using the Naive Bayes classifier implemented in the QIIME 2 q2-feature-classifier plugin [29], trained on the PR2 database v5.0.1 [30] with a 99% similarity threshold for species-level classification.

### Statistical analysis

All statistical analyses were performed using R software v4.3.1 [31]. Nematode OTU counts were converted to relative abundances (*i.e.,* the number of OTUs assigned to a taxon divided by the total OTUs per sample) prior to statistical analyses. Alpha diversity metrics were calculated using rarefied data. Analyses were performed using linear mixed-effects models. Soil type, extraction type, and primer set were included as fixed effects, and subplot was included as a random effect. Significance was assessed using analysis of variance (ANOVA) (*lmerTest*), and significant effects were followed by pairwise comparisons using estimated marginal means (*emmeans*). Model assumptions were evaluated using standard residual diagnostics, including residuals versus fitted values (linearity and homoscedasticity), and Q–Q plots of residuals (normality). When departures from normality and/or unequal variance were observed, response variables were log- or square-root transformed as appropriate and the models were subsequently refitted. OTU counts were assigned to nematode feeding groups (e.g., herbivores, fungivores, bacterivores, omnivores, and predators), ecological indices (maturity index (MI), enrichment index (EI) and structure index (SI)), and c-p/p-p (colonizer-persister) classes using the Nematode Indicator Joint Analysis (NINJA) tool [32], accessed on December 2, 2024 (https://shiny.wur.nl/ninja/). The effects of DNA source, extraction type and primer choice on nematode community composition were analyzed using ordination and permutation-based multivariate analyses. Community dissimilarities were calculated using a Bray-Curtis dissimilarity matrix [33]. Nonmetric multidimensional scaling (NMDS) was used to visualize differences in community composition. Permutational multivariate analysis of variance (PERMANOVA; 9,999 permutations) was conducted to test for differences between DNA sources within each primer set. Differences among primer sets (NF1, NemF, and NemFopt) within each soil type were assessed using analysis of similarities (ANOSIM; 999 permutations). Principal coordinates analysis (PCoA) was used as a complementary ordination to visualize community patterns, and homogeneity of multivariate dispersion was evaluated using PERMDISP (betadisper) with permutation tests and pairwise comparisons.

## Results

### DNA sources influence on primer specificity

**Fig 1:**
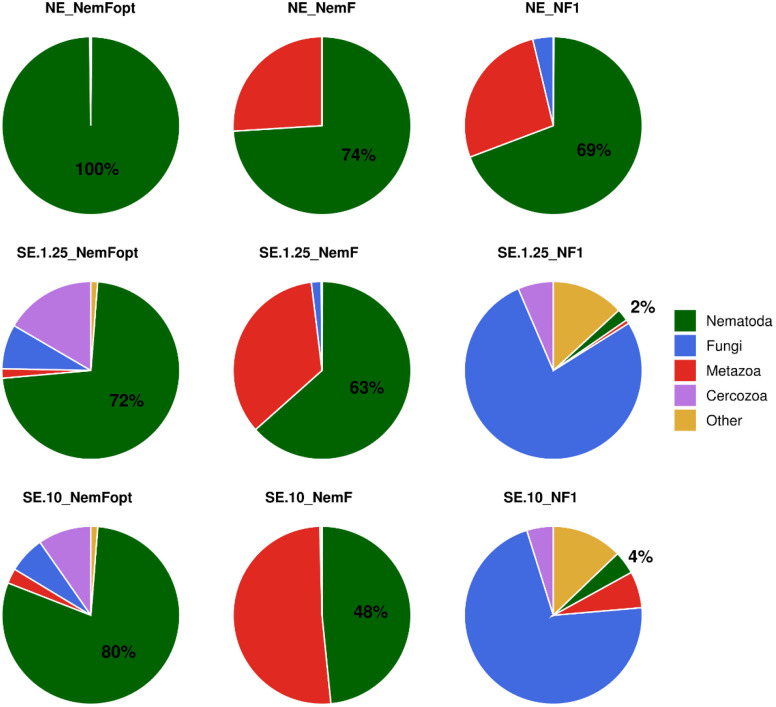
Mean relative abundance of nematode sequence reads amplified using different primer sets and DNA sources at phylum rank. NE = Nematodes DNA extraction, SE = Soil DNA extraction, NemFopt = NemFopt–18Sr2bRopt, NemF = NemF–18Sr2b, and NF1 = NF1–18Sr2b. SE.10 = Soil DNA extraction using 10 g of dry soil, and SE.1.25 = Soil DNA extraction using 1.25 g of dry soil.

The relative abundance of nematode sequence varied significantly across primer types and DNA sources (Fig 1). When NE was used, all primer sets yielded high proportions of nematode reads, with NemFopt producing the highest (100%), followed by NemF (74%) and NF1 (69%). In contrast, SE showed greater variation in recovering nematode reads. The NemFopt primer pair captured the highest proportions, detecting 72% and 80% nematode reads from 1.25 g and 10 g of dry soil samples, respectively. NemF captured the second highest proportions, with 63% from 1.25 g and 48% from 10 g of dry soil samples. The NF1 primer set had the least detection on soil DNA, recovering only 2% and 4% nematode reads from the 1.25 g and 10 g samples, respectively. Non-target sequences, primarily from fungi, metazoans, and cercozoans, were more prominent in soil DNA extractions, especially when using the NF1 primer. Overall, these results indicate that both primer selection and DNA source substantially influence the recovery of nematode sequences.

### Taxonomic resolution and community composition

The performance of the NF1, NemF, and NemFopt primer sets in amplifying and detecting nematodes varied across DNA extraction methods. A total of 51 nematode genera representing 27 families were identified (S1 Table). The NemFopt and NemF primers detected a higher number of nematode families compared to NF1. The NF1 primer failed to capture several key families including Aguinidae, Aphelenchidae, Aphelenchoididae, Belondiridae, and Hoplolaimidae in both extraction methods. Cephalobidae, Plectidae, Pratylenchidae and Rhabditidae were among the most abundant nematode families across all treatments (S1 Table).

**Fig 2:**
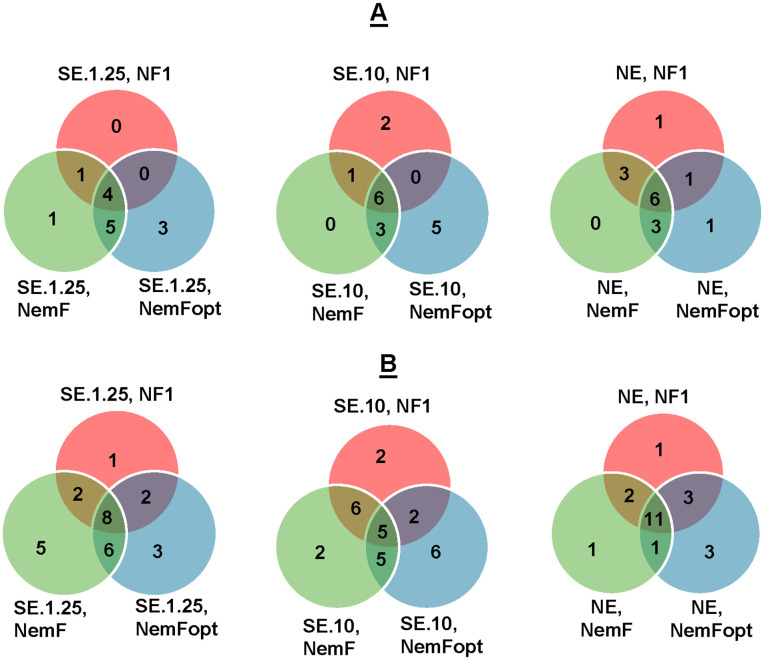
Venn diagrams showing the number of nematode taxa detected using different primers and DNA sources. **(A)** Identification at the genus level, and **(B)** Identification at the species level. NE = Nematodes DNA extraction, SE = Soil DNA extraction, NemFopt = NemFopt–18Sr2bRopt, NemF = NemF–18Sr2b, and NF1 = NF1–18Sr2b. SE.10 = Soil DNA extraction using 10 g of dry soil and SE.1.25 = Soil DNA extraction using 1.25 g of dry soil.

Similar trends were observed at the genus and species levels (Fig 2). The NF1 primer detected fewer taxa overall, whereas NemFopt identified a higher number of taxa across all extraction types (Fig 2A). At the species level, NE samples amplified with NemFopt showed the highest overlap with those amplified using NemF (Fig 2B).

**Fig 3:**
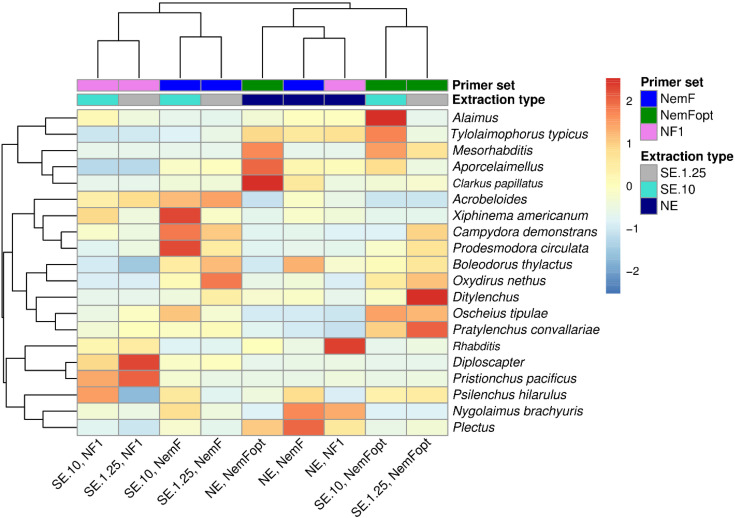
Hierarchical clustering heat map based on the relative abundance of the 20 most abundant nematode taxa. Columns represent DNA extraction method and rows correspond to nematodes OTUs assigned at the genus and species levels. Dendrograms of hierarchical cluster analysis, grouping nematode genera and DNA extraction methods, are displayed on the left and top, respectively. Colors indicate standardized abundance values, with warmer colors representing higher relative abundance. NE = Nematodes DNA extraction, SE = Soil DNA extraction, NemFopt = NemFopt–18Sr2bRopt, NemF = NemF–18Sr2b, and NF1 = NF1–18Sr2b. SE.10 = Soil DNA extraction using 10 g of dry soil, and SE.1.25 = Soil DNA extraction using 1.25 g of dry soil.

Distinct patterns of nematode detection were observed among the primer sets (Fig 3). All primers identified similar nematode communities when using the NE approach. Notably, NemFopt also detected nematodes communities from SE that closely clustered with those from NE, indicating its superior performance in capturing representative community profiles across both extraction methods.

**Fig 4:**
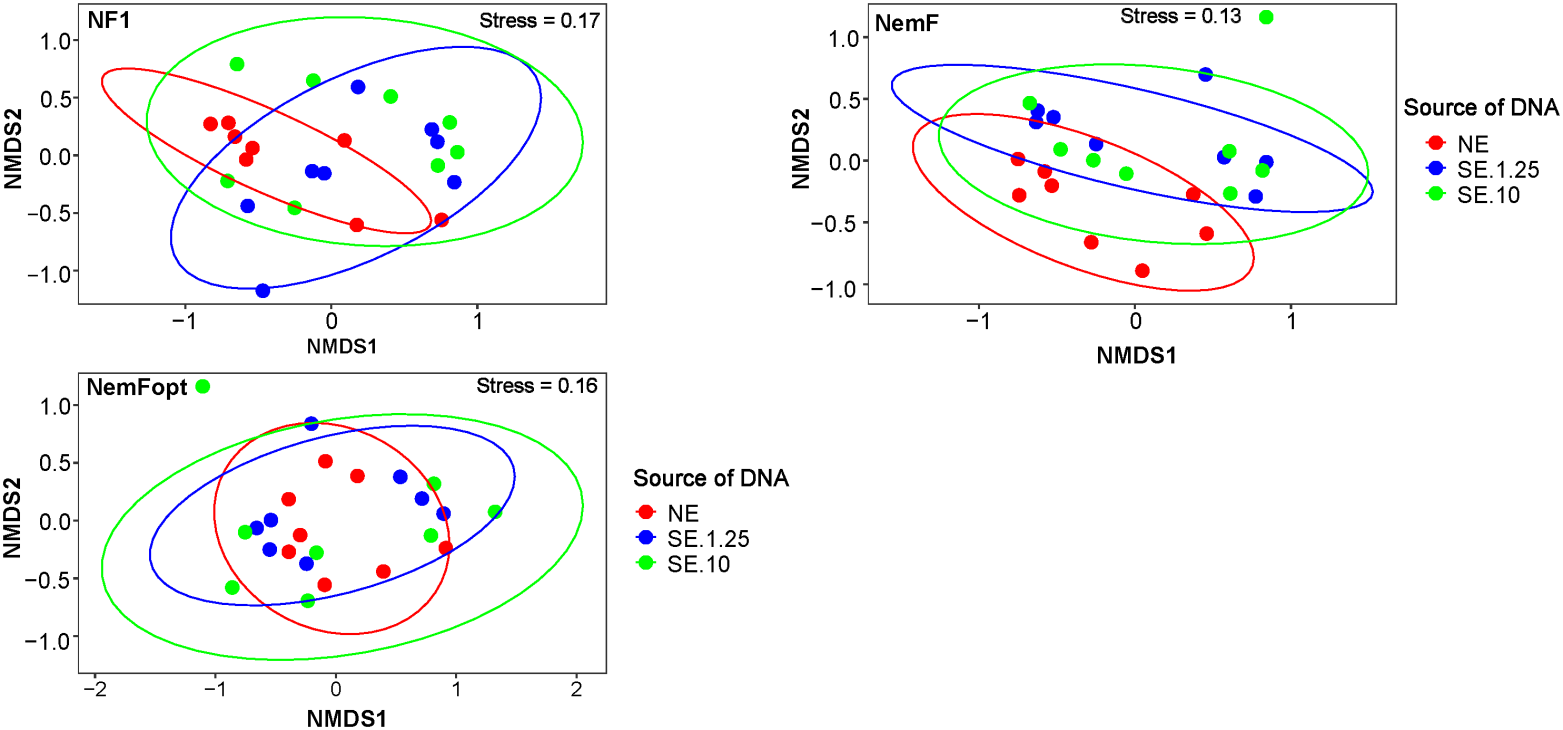
Non-metric multidimensional scaling (NMDS) ordination plot illustrating differences among DNA sources plotted in species space across primer sets (NF1, NemF, and NemFopt). The circles surrounding the clusters represent 90% confidence intervals. Each point represents a sample, and colors indicate extraction method. NE = Nematodes DNA extraction, SE = Soil DNA extraction, NemFopt = NemFopt–18Sr2bRopt, NemF = NemF–18Sr2b, and NF1 = NF1–18Sr2b. SE.10 = Soil DNA extraction using 10 g of dry soil, and SE.1.25 = Soil DNA extraction using 1.25 g of dry soil.

NMDS and PERMANOVA analyses revealed significant overall differences (P < 0.001) in nematode community composition among primer sets and DNA sources, indicating that both factors influence community detection (Fig 4). For NemFopt, PERMANOVA detected significant differences among DNA sources (P = 0.02), however, pairwise comparisons showed no significant difference between NE and SE.10 (F = 0.62, P = 0.77), consistent with the clustering patterns observed in Fig 3.

**Fig 5:**
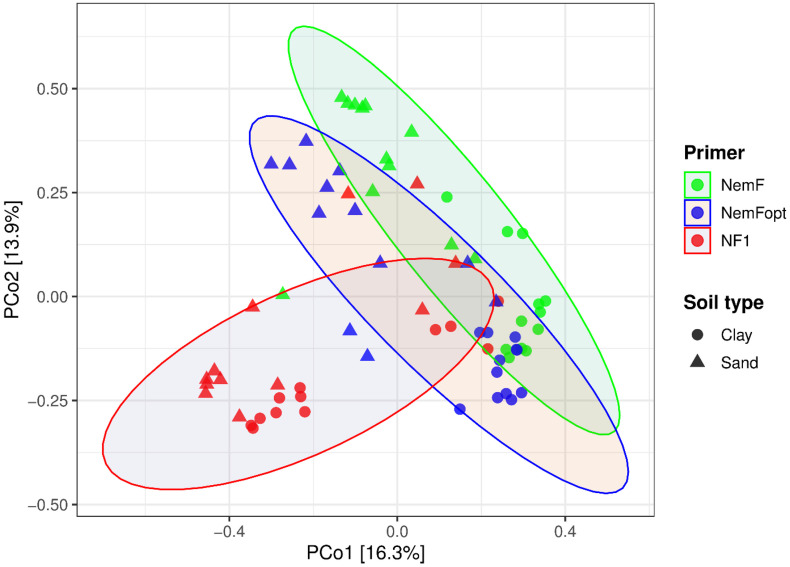
Principal coordinates analysis (PCoA) of nematode community composition based on NF1, NemF, and NemFopt primers across soil types. Points represent individual samples, colored by primer and shaped by soil type (clay vs. sand). Ellipses indicate 95% confidence intervals around primer-specific centroids. Axis percentages denote the proportion of variance explained by each principal coordinate.

PCoA revealed a clear separation of nematode communities by primer set, with NF1 forming a distinct cluster relative to NemF and NemFopt (Fig 5). Within each soil type, ANOSIM confirmed significant differences among primer sets. In sandy soils, pairwise comparisons indicated moderate dissimilarity among primer pairs (R = 0.29–0.47, P ≤ 0.002), whereas stronger dissimilarities were observed in clay soils (R = 0.43–0.55, P = 0.001). PERMDISP revealed significant differences in multivariate dispersion among primer groups (F = 9.79, P = 0.001). Pairwise tests showed that NemF differed significantly in dispersion from both NF1 and NemFopt, while NF1 and NemFopt did not differ significantly. Collectively, these results indicate that primer effects reflect both shifts in community composition and differences in within-group variability.

### Functional and ecological insight

**Fig 6:**
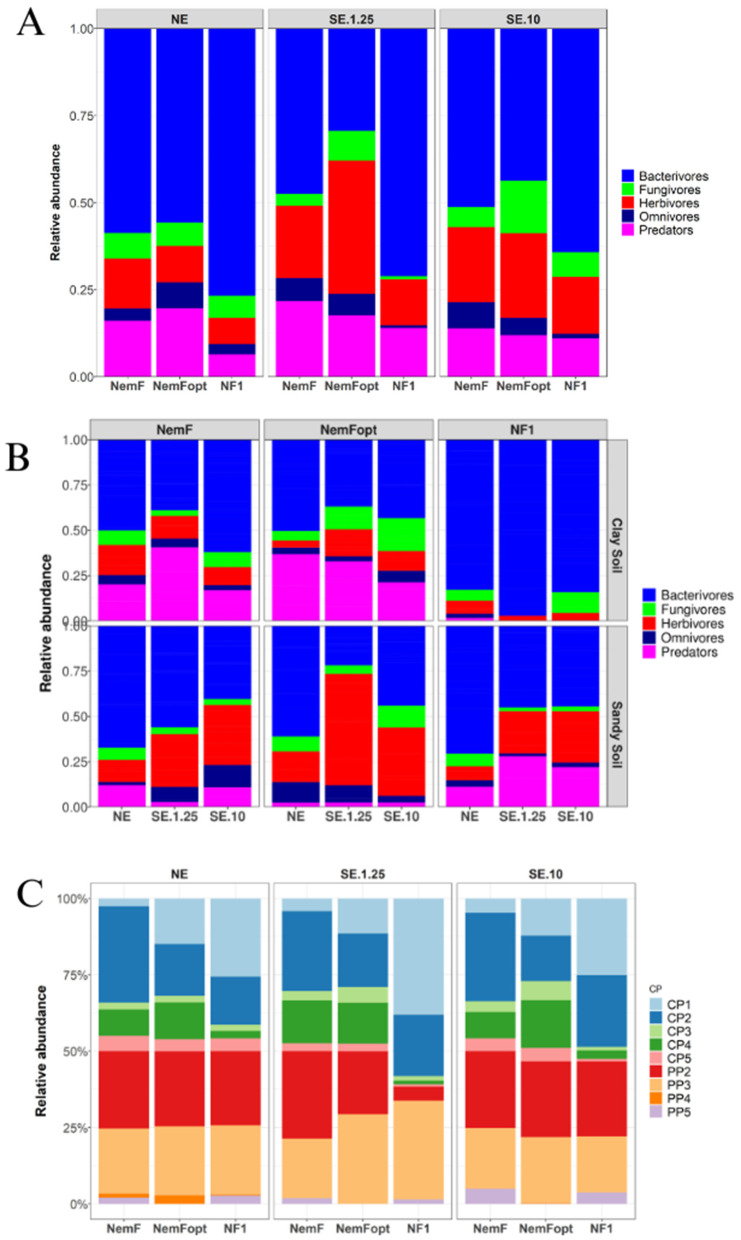
Relative abundance of nematode communities. **(A)** Relative abundance of feeding groups across different DNA sources using the three primer sets, **(B)** Relative abundance of feeding groups in different agricultural soils, and **(C)** Relative abundance of nematode colonizer-persister (c-p) classification, pp is used to differentiate plant-parasitic nematodes from free-living nematodes. NE = Nematodes DNA extraction, SE = Soil DNA extraction, NemFopt = NemFopt–18Sr2bRopt, NemF = NemF–18Sr2b, and NF1 = NF1–18Sr2b.

The distribution of nematode feeding groups varied across primer pairs, soil types and DNA sources (Table 2). All primers consistently identified bacterivores as the most abundant feeding group, accounting for over 58% of the total feeding groups (Fig 6A,B). Predators and omnivores were more frequently recovered from SE using the NF1 primer, whereas fungivores showed variable representation across DNA sources, primers and soil types. When comparing soil types, the NF1 primer revealed a higher abundance of bacterivores in clay soil than in sandy soil, regardless of DNA extraction method (88% vs. 53%; Fig 6B). Conversely, the primer showed higher herbivore abundance in sandy soil compared to clay soil (20% vs. 5%). Omnivores and predators were not detected in SE using the NF1 primer. The NemF and NemFopt primers captured a higher relative abundance of bacterivores in sandy soil than in clay soil for NE samples, while bacterivore abundance varied across soil types in SE samples. NemFopt also detected a higher relative abundance of herbivores in sandy soil than in clay soil.

**Table 2:** Summary of analyses of variance (F value) for the effects of soil type (Soil), DNA extraction method (Extraction), and their interaction (Soil × Extraction) on nematode variables, as determined by each primer pair. Two soil types (clay and sandy), and three DNA sources (NE, SE.1.25 and SE.10) were compared across the primers NF1, NemF, and NemFopt.

Variable	Soil	Extraction	Primer	Soil x Extraction	Soil x Primer	Primer x Extraction	Soil x Extraction x Primer
Ba	4.27*	2.86	10.19***	2.43	5.68**	0.67	1.10
Fu	1.07	1.36	1.85	0.79	0.06	0.57	0.31
He	38.84***	7.20**	5.41**	6.82**	3.08*	2.14	0.65
Om	2.17	0.00	2.14	0.09	0.11	0.40	1.00
Pr	7.18**	0.07	1.82	1.47	21.18**	0.52	1.91
Shannon	1.39	0.53	3.09	0.51	4.45*	1.48	0.60
MI	0.36	0.19	20.21***	1.22	1.69	0.63	0.98
EI	21.75***	1.16	18.51***	0.80	0.48	0.32	0.81
SI	0.98	1.29	17.52	2.08	0.12	2.58*	1.36

Ba = relative abundance of bacterivores, Fu = relative abundance of fungivores, He = relative abundance of herbivores, Om = relative abundance of omnivores, Pr = relative abundance of predators,

*, ** and *** denote significant effects at P < 0.05, P < 0.01 and P < 0.001 levels, respectively.

MI = maturity index, EI = enrichment index, SI = structure index.

The colonizer-persister (c-p) classification varied depending on the primer and DNA source used (Fig 6C). NE samples consistently yielded higher proportions of cp-1, cp-2 taxa, pp-2 and pp-3. Conversely, SE samples had more balanced cp distributions. Notably, NemFopt failed to detect pp-5 across all DNA sources, indicating that primer choice and DNA source can influence ecological interpretations.

### Nematode diversity indices

**Fig 7:**
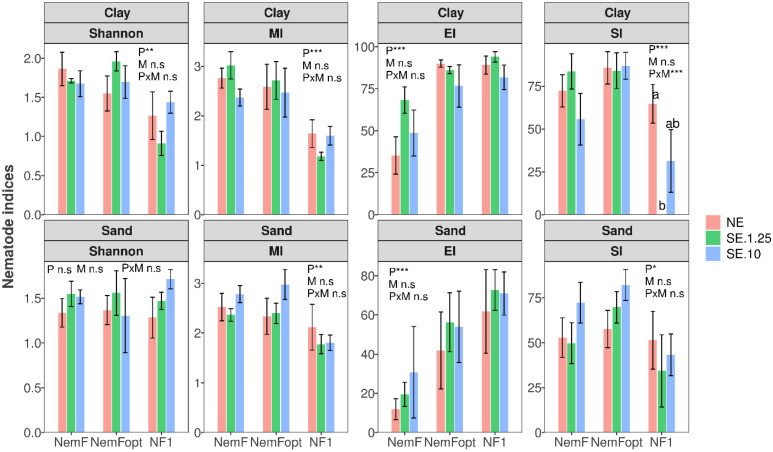
Nematode indices using different primer sets and DNA sources. Differences among extraction methods and primer sets were assessed using linear mixed-effects models with subplot as a random effect. MI = maturity index, EI = enrichment index, SI = structure index. NE = Nematodes DNA extraction, SE = Soil DNA extraction, NemFopt = NemFopt–18Sr2bRopt, NemF = NemF–18Sr2b, and NF1 = NF1–18Sr2b. P = Primer set, M = Method of extraction and their interactions. ANOVA results: ***P < 0.001, **P < 0.01, *P < 0.05, n. s. = not significant.

Rarefaction curves based on sequencing depth indicated that the sampling effort was sufficient to capture the majority of nematode diversity across all DNA sources and primer sets. Ecological indices such as the MI, EI, and SI varied with soil type and DNA source (Table 2, Fig 7). Across both clay and sandy soils, ecological indices were primarily influenced by primer choice rather than soil input volume. The MI differed significantly among primer sets in both soil types (P < 0.01), whereas soil volume and primer × volume interactions were not significant. Similarly, the EI exhibited significant primer effects in both clay and sandy soils (P < 0.001), with no significant influence of soil volume. In contrast, the SI was more sensitive to methodological variation. SI differed significantly among primers in both clay and sandy soils (P < 0.05), and significant differences were also observed between soil input volumes for NF1 in clay soils (Fig 7).

## Discussion

For nematode ecological studies, soil DNA extraction has historically been underutilized, primarily due to low nematode sequence recovery [11,19]. The commonly used NF1–18Sr2b (NF1) primer targets the 18S rRNA V6–V8 region and has been widely applied, but it is not nematode-specific and co-amplifies non-target organisms [6,13]. Consequently, NF1 often yields only ~4–10% nematode sequences in soil DNA extractions, compared to over 80% from nematode DNA extraction [13,18,19]. This poor recovery has contributed to the perception that small soil subsamples cannot capture complete nematode communities. For example, Griffiths et al., [18] reported higher nematode richness from pre-isolated samples than from bulk soil, although this method is labor-intensive and may introduce extraction-related biases. However, recent large-scale studies suggest that these limitations are methodological rather than fundamental [19]. They showed that nematode communities recovered using soil-extracted DNA were broadly comparable to those obtained from nematode-sample DNA across a continent-scale survey. The key challenges in soil DNA approaches are now understood to be the use of non-specific primers and small soil input volumes. The former causes most reads to derive from non-nematode soil biota, while the latter limits the detection of rare nematode taxa. For instance, Kawanobe et al., [34] demonstrated that conventional 18S primers amplify abundant non-nematode sequences and that small soil quantities reduce sensitivity to rare taxa, advocating the need for nematode-specific primers to overcome these issues. Therefore, improving primer design and increasing soil mass can substantially improve nematode recovery.

To evaluate this further, two nematode-targeted degenerate primer pairs, NemF/18Sr2b (NemF) and NemF/18Sr2bRopt (NemFopt), were tested using both nematode-enriched DNA extraction (NE) and direct soil DNA extraction (SE) methods. As expected, NE samples yielded a higher proportion of nematode reads than SE samples, however, primer choice had a stronger effect than DNA source. For example, the NF1 primer performed poorly, detecting only 2–4% of nematode sequences in SE samples, confirming previous studies [13,23]. In contrast, the degenerate NemF and NemFopt primers greatly improved nematode detection from SE. Using NemF, the majority of reads in 1.25 g soil extractions were nematode-derived, aligning with findings by Sikder et al., [24], who obtained ~74% of nematode sequences from SE extracts. The NemFopt primer yielded the highest fractions of nematode reads (>70% of total sequences) in SE, outperforming the reference NF1 primer. While Waeyenberge et al., [14], had cautioned that NemFopt might co-amplify non-target organisms and suggested it for broad eukaryotic profiling, our results indicate otherwise. NemFopt was highly selective for nematodes, yielding 100% of nematode reads in NE and over 70% in SE samples. These findings confirm our hypothesis that nematode-specific primers substantially enhance both the sensitivity and specificity of soil DNA metabarcoding, supporting the use of soil DNA extraction as a reliable alternative to nematode isolation [13,21,23].

Differences in DNA extraction methods had a pronounced effect on nematode detection. The SE outperformed NE in capturing family-level diversity, and uniquely detected four families (Bunonematidae, Rhabdolaimidae, Aphelenchidae, Tylenchidae) that NE missed. In contrast, only a single family (Panagrolaimidae) was unique to NE. NE-derived samples showed high relative abundance of certain families, such as Nygolaimidae and Plectidae, suggesting that extraction method can bias community composition. Similar results have been reported in prior comparisons of bulk nematode DNA versus soil DNA methods [18,19]. More importantly, direct soil extractions tend to underrepresent some predators while overrepresenting other groups. This shows the importance of choosing an appropriate extraction method for accurate nematode biodiversity estimates.

Likewise, primer choice greatly influenced which nematodes were detected, signifying primer-driven biases in metabarcoding. In this study, the NemF and NemFopt consistently amplified taxa such as *Anaplectus* sp., *Ditylenchus* sp., and *Oxydirus nethus* across all extraction methods, whereas NF1 failed to recover these taxa. This discrepancy likely results from primer–template mismatches. For example, *Anaplectus* sp. went undetected by NF1 in SE samples, even though Ahmed et al., (11) successfully recovered it from a mock community using the same primer. The difference is that in our soil communities, the NF1 binding site in *Anaplectus* probably contained nucleotide mismatches, preventing amplification, whereas in controlled mock communities the primer perfectly matched the target sequences. In contrast, NemF and NemFopt target a more conserved region of the 18S rRNA, enabling broader taxonomic coverage. The NemF primer was specifically designed for enhanced nematode specificity and coverage, allowing amplification of a wide range of nematodes while avoiding non-target DNA. The success of NemF and NemFopt in detecting *Anaplectus* and other taxa missed by NF1underscores the importance of using updated, nematode-tailored primers for accurate metabarcoding studies.

Overall, the NE method using NemF and NemFopt primer pairs yielded fewer taxa than the SE approach. This is likely due to the limited extraction efficiency of the DNeasy Blood and Tissue Kit. Despite pre-treatment with liquid nitrogen [14], intact nematodes were still observed microscopically, suggesting incomplete lysis. In contrast, the DNeasy PowerMax Soil Kit and DNeasy PowerSoil Pro Kit produced higher DNA quality and concentrations (25 ng/µL and 35 ng/µL, respectively) compared to the DNeasy Blood and Tissue Kit (4 ng/µL). These results suggest that future nematode DNA extraction protocols could benefit from incorporating soil DNA extraction kits, as supported by a recent study [35]. Furthermore, nematode DNA extraction remains widely accepted in metabarcoding studies [6,11], and continues to serve as a benchmark for evaluating primer performance in high-throughput sequencing. Our results confirm that a highly specific primer should be able to detect similar nematode communities across both SE and NE methods. This was validated through NMDS and cluster analyses, where NemFopt, particularly in SE.10, identified nematode assemblages comparable to those obtained from NE samples. These findings suggest that NemFopt enhances nematode detection in soil DNA extracts and yields community profiles closely matching those derived from isolated nematodes. Moreover, current soil sampling practices are considered inefficient, as large amounts of soil, often exceeding 10 kg per site, are collected from the field, while only a small fraction (typically <200 g) is used for analysis [6,35]. Validating and optimizing soil DNA extraction methods could therefore reduce resource waste, streamline workflows, and improve the efficiency and accuracy of nematode metabarcoding studies.

In the present study, nematode diversity patterns further supported the effects of primer choice and extraction method. OTU richness was higher in the SE approach than in the NE when using the NemF and NemFopt primers. Conversely, NF1 yielded higher richness in NE across all DNA sources. The finding of NF1 is consistent with Donhauser et al (19), but the authors attributed the lower richness in soil DNA extractions to the small soil volumes used (10 g). However, our results with the NemF and NemFopt indicate that 10 g of dry soil was sufficient to capture the local nematode community when nematode-specific primers were used.

A key ecological insight from this study is that primer choice can shape perceived nematode community structure without fundamentally altering higher‐order ecological interpretations. Across both soil types, the ecological indices were largely stable between NE and SE, and this stability persisted regardless of the soil input volume used for SE (1.25 vs 10 g of dry soil). For both the MI and EI, no significant differences were detected between SE (at either soil volume) and NE, indicating that ecological inferences based on these indices are robust to DNA source and within the tested range of soil input masses. These findings directly support our hypothesis that comparable community-level ecological signals can be obtained from different extraction inputs and soil types and further suggest that increasing soil mass for soil DNA extraction does not necessarily provide additional ecological information, at least as reflected by MI and EI. In contrast, the SI was the only index that showed sensitivity to methodological differences, with a significant effect of extraction method (DNA source) observed with NF1. This likely reflects the strong dependence of SI on accurate recovery of higher trophic levels (e.g., omnivores and predators), which are typically rare and therefore more prone to under-detection or under-representation [6,10]. This was particularly evident with NF1, which underrepresented these groups in clay soils.

The absence of soil volume effects on community composition and ecological indices suggests that increasing soil input does not necessarily enhance nematode metabarcoding outcomes, supporting previous work demonstrating consistent performance using ≤0.25 g of soil [24]. This finding highlights the importance of protocol efficiency and primer optimization over bulk soil quantity and has clear implications for methodological standardization across laboratories. The adoption of optimized primers such as NemFopt, along with harmonized DNA extraction protocols, consistent soil input masses, and benchmarking using mock communities, would substantially improve cross-study comparability. We acknowledge that this study was limited to two soil types and field sites, which may constrain generalization to other soil contexts. Future work should evaluate a broader range of soil systems, larger or replicated soil inputs to improve detection of rare taxa, and alternative 18S rRNA regions to further refine primer performance and establish robust standards for nematode metabarcoding.

Community analyses consistently indicate that DNA source and primer choice interact to influence perceived nematode community composition, but that this effect is minimized when communities are recovered using NemFopt. NMDS ordinations showed that only NemFopt produced similar community structures across DNA sources, irrespective of soil input volume, indicating strong methodological consistency. In contrast, NF1 and NemF displayed greater separation among DNA sources, suggesting higher sensitivity to extraction-related variation. Principal coordinates analysis further demonstrated that primer choice structured community composition more strongly than soil type, with distinct clustering among primer sets, however, NemFopt-derived communities remained broadly comparable to those detected by both NF1 and NemF. Heatmap clustering reinforced these patterns, showing that NE communities clustered together regardless of primer set, consistent with previous reports that NE yields more homogeneous community structure than SE [19]. NemFopt-derived SE communities clustered closely with NE, indicating that this primer mitigates extraction-related bias and enhances cross-method comparability. Finally, downstream choices such as sequencing pipelines, reference databases, and bioinformatics filters may also influence nematode metabarcoding results. Therefore, there is a need for standardization of nematode metabarcoding workflows, including testing of new primers, DNA extraction protocols and reference databases, to ensure reproducibility and improve cross-study comparability. While the PR2 database was employed for taxonomic assignment and primer validation because of its broad coverage of eukaryotic diversity, we acknowledge that it may not provide the same level of curation for nematodes as specialized databases such as NemaBase or NemaTaxa. Consequently, taxonomic resolution at lower ranks (e.g., genus or species) may be limited and potentially prone to misassignment.

## Conclusion

Metabarcoding has become an essential tool in nematode ecological studies, however, challenges remain that underscore the need for methodological standardization. Although several primers have been designed, further evaluation is necessary to determine the most suitable primers for nematode metabarcoding, similar to standardization efforts already undertaken in other fields such as microbial and fungal ecology. Comparative studies across different soil types, primer sets, and extraction methods are crucial for improving reliability and promoting broader adoption of this technique.

Our findings demonstrate that primer choice strongly influences nematode identification, feeding group composition, and diversity indices. Degenerate primers such as NemFopt provided greater taxonomic coverage in both soil and nematode DNA extractions compared to the widely used NF1 primer. Contrary to concerns that soil DNA extraction may not generate reliable nematode profiles due to limited sample volumes, NemFopt applied to SE.10 successfully captured communities comparable to those obtained through nematode DNA extraction. Our study further demonstrates that soil DNA input volume within the tested range does not substantially influence nematode community inference. Ecological indices were generally consistent between nematode- and soil-extracted DNA, except for SI. Community composition was sensitive to primer choice, but NemFopt reduced extraction-related variability. The study was limited to two soil types, two field sites, and three primer sets. Further work should assess a broader range of soil conditions, larger or replicated soil inputs, and alternative 18S rRNA regions. The findings in this study highlight the importance of rigorous primer evaluation using field-derived soil samples and support ongoing efforts to standardize primer selection and soil DNA extraction protocols. Adoption of such standardized approaches will enhance the reliability, reproducibility and interpretability of nematode ecological studies and soil health assessments.

## Supporting information

S1 Table The relative abundance of nematode families determined from nematode DNA extraction (NE) and soil DNA extraction (SE). (DOCX)
