# Supplementary material for: DNA source and primer choice affect the reliability of metabarcoding for nematode community profiling in agricultural soils
Source: PLoS One. 2026 Apr 10;21(4):e0344569. doi: 10.1371/journal.pone.0344569 (PMC13068266; doi:10.1371/journal.pone.0344569)
Supplement: S1 Table — (DOCX) [file pone.0344569.s001.docx]

**S1 Table. The relative abundance of nematode families determined from nematode DNA extraction (NE) and soil DNA extraction (SE).**

|  | **NemF** | | | **NemFopt** | | | **NF1** | | |
| --- | --- | --- | --- | --- | --- | --- | --- | --- | --- |
| **Family** | **SE.1.25** | **SE.10** | **NE** | **SE.1.25** | **SE.10** | **NE** | **SE.1.25** | **SE.10** | **NE** |
| Alaimidae | 0.05±0.05 | 0.32±0.23 | 2.56±1.26 | 0.31±0.31 | 11.86±11.86 | 1.34±0.70 | 1.02±1.02 | 3.29±2.41 | 2.48±1.48 |
| Anguinidae | 1.37±0.54 | 0.72±0.36 | 0.83±0.35 | 3.15±1.22 | 0.51±0.47 | 0.41±0.29 | 0.00±0.00 | 0.00±0.00 | 0.00±0.00 |
| Aphelenchidae | 0.22±0.22 | 0.04±0.04 | 0.00±0.00 | 0.41±0.41 | 0.23±0.23 | 0.00±0.00 | 0.00±0.00 | 0.00±0.00 | 0.00±0.00 |
| Aphelenchoididae | 0.11±0.11 | 0.00±0.00 | 0.00±0.00 | 2.15±1.19 | 1.86±0.93 | 0.32±0.19 | 0.00±0.00 | 0.00±0.00 | 0.00±0.00 |
| Aporcelaimidae | 1.53±0.79 | 1.22±0.53 | 1.61±0.88 | 1.37±0.66 | 2.52±1.09 | 3.65±2.48 | 0.00±0.00 | 0.00±0.00 | 1.46±0.88 |
| Belondiridae | 19.12±8.1 | 7.27±4.73 | 3.63±1.55 | 14.17±6.37 | 9.87±5.74 | 2.02±0.89 | 0.00±0.00 | 0.00±0.00 | 0.00±0.00 |
| Bunonematidae | 0.59±0.41 | 0.00±0.00 | 0.00±0.00 | 0.61±0.44 | 0.00±0.00 | 0.00±0.00 | 0.07±0.07 | 0.00±0.00 | 0.00±0.00 |
| Campydoridae | 3.55±3.55 | 5.12±5.12 | 0.3±0.3 | 3.41±3.41 | 0.00±0.00 | 0.19±0.19 | 0.75±0.75 | 1.25±1.25 | 0.07±0.07 |
| Cephalobidae | 10.51±3.77 | 9.27±3.42 | 3.96±2.05 | 0.08±0.08 | 0.06±0.05 | 0.05±0.04 | 7.84±3.00 | 6.00±20.39 | 2.1±1.23 |
| Diphtherophoridae | 1.58±1.28 | 1.25±1.18 | 3.18±3.18 | 1.69±1.38 | 4.73±4.40 | 3.56±3.56 | 1.01±1.01 | 0.92±0.79 | 3.37±3.37 |
| Diplopeltidae | 0.90±0.46 | 0.00±0.00 | 0.26±0.17 | 1.14±0.73 | 0.82±0.82 | 0.06±0.06 | 0.00±0.00 | 0.00±0.00 | 0.07±0.07 |
| Hoplolaimidae | 0.00±0.00 | 0.00±0.00 | 0.00±0.00 | 1.90±1.55 | 0.79±0.79 | 0.08±0.08 | 0.00±0.00 | 0.00±0.00 | 0.00±0.00 |
| Longidoridae | 0.57±0.57 | 3.00±3.00 | 0.53±0.53 | 0.00±0.00 | 0.04±0.04 | 0.00±0.00 | 0.25±0.25 | 1.55±1.55 | 0.32±0.32 |
| Microlaminated | 1.85±1.04 | 4.50±3.92 | 0.19±0.16 | 2.15±0.84 | 0.83±0.79 | 0.05±0.05 | 0.41±0.41 | 0.00±0.00 | 0.08±0.08 |
| Monhysteridae | 0.21±0.15 | 0.47±0.35 | 0.09±0.09 | 0.22±0.18 | 0.37±0.26 | 0.19±0.19 | 0.85±0.46 | 3.08±1.62 | 0.12±0.12 |
| Mononchidae | 1.17±0.54 | 1.23±0.66 | 6.51±2.80 | 2.24±1.09 | 1.60±0.97 | 17.16±7.90 | 0.00±0.00 | 0.00±0.00 | 0.6±0.4 |
| Mylonchulidae | 0.02±0.02 | 0.21±0.14 | 0.23±0.16 | 0.00±0.00 | 0.42±0.31 | 0.43±0.24 | 0.00±0.00 | 0.00±0.00 | 0.34±0.21 |
| Neodiplogasteridae | 0.00±0.00 | 0.76±0.76 | 0.00±0.00 | 0.20±0.11 | 0.00±0.00 | 0.00±0.00 | 6.67±3.78 | 4.96±2.58 | 0.29±0.29 |
| Nygolaimidae | 0.92±0.47 | 3.58±2.31 | 5.7±4.69 | 0.00±0.00 | 0.00±0.00 | 0.00±0.00 | 0.67±0.67 | 1.09±0.81 | 4.86±4.12 |
| Panagrolaimidae | 0.00±0.00 | 0.00±0.00 | 0.00±0.00 | 0.00±0.00 | 0.00±0.00 | 0.00±0.00 | 0.00±0.00 | 0.00±0.00 | 0.01±0.01 |
| Plectidae | 1.33±0.90 | 2.68±1.44 | 9.90±3.60 | 2.37±1.17 | 2.85±1.04 | 6.85±2.67 | 0.00±0.00 | 1.34±0.72 | 5.28±2.14 |
| Pratylenchidae | 5.48±2.90 | 4.95±2.41 | 2.04±1.17 | 12.77±6.23 | 9.38±4.61 | 2.82±1.53 | 6.07±3.01 | 4.33±2.08 | 1.72±1.06 |
| Rhabditidae | 1.28±0.46 | 1.29±0.56 | 0.79±0.33 | 2.95±1.13 | 3.5±1.69 | 5.23±2.16 | 8.68±2.9 | 5.55±1.96 | 9.27±3.65 |
| Rhabdolaimidae | 0.17±0.13 | 0.04±0.04 | 0.00±0.00 | 0.03±0.03 | 0.00±0.00 | 0.00±0.00 | 0.2±0.2.00 | 0.65±0.36 | 0.00±0.00 |
| Trichodoridae | 0.00±0.00 | 0.00±0.00 | 0.69±0.69 | 0.00±0.00 | 0.19±0.19 | 2.4±2.40 | 0.00±0.00 | 0.00±0.00 | 0.07±0.07 |
| Tylenchidae | 1.09±0.51 | 1.22±0.49 | 1.58±0.63 | 1.32±0.44 | 1.62±0.65 | 0.62±0.23 | 0.07±0.07 | 1.46±0.74 | 0.91±0.37 |
| Tylenchulidae | 0.15±0.15 | 0.00±0.00 | 0.00±0.00 | 0.00±0.00 | 0.00±0.00 | 0.00±0.00 | 0.00±0.00 | 0.58±0.39 | 0.00±0.00 |

Values are presented as mean ± standard error (SE).

NemFopt = NemFopt–18Sr2bRopt, NemF = NemF–18Sr2b, and NF1 = NF1–18Sr2b.

SE.10 = Soil DNA extraction using 10 g of dry soil, and SE.1.25 = Soil DNA extraction using 1.25 g of dry soil.
